# Biocompatibility between Silicon or Silicon Carbide surface and Neural Stem Cells

**DOI:** 10.1038/s41598-019-48041-3

**Published:** 2019-08-08

**Authors:** Gabriele Bonaventura, Rosario Iemmolo, Valentina La Cognata, Massimo Zimbone, Francesco La Via, Maria Elena Fragalà, Maria Luisa Barcellona, Rosalia Pellitteri, Sebastiano Cavallaro

**Affiliations:** 1 Institute for Biomedical Research and Innovation, Italian National Research Council, Catania, Italy; 2 Institute for Microelectronics and Microsystems, Italian National Research Council, Catania, Italy; 3 Department of Chemical Sciences, University of Catania, Catania, Italy; 4 Department of Pharmaceutical Sciences, University of Catania, Catania, Italy

**Keywords:** Neuroscience, Biomaterials

## Abstract

Silicon has been widely used as a material for microelectronic for more than 60 years, attracting considerable scientific interest as a promising tool for the manufacture of implantable medical devices in the context of neurodegenerative diseases. However, the use of such material involves responsibilities due to its toxicity, and researchers are pushing towards the generation of new classes of composite semiconductors, including the Silicon Carbide (3C-SiC). In the present work, we tested the biocompatibility of Silicon and 3C-SiC using an *in vitro* model of human neuronal stem cells derived from dental pulp (DP-NSCs) and mouse Olfactory Ensheathing Cells (OECs), a particular glial cell type showing stem cell characteristics. Specifically, we investigated the effects of 3C-SiC on neural cell morphology, viability and mitochondrial membrane potential. Data showed that both DP-NSCs and OECs, cultured on 3C-SiC, did not undergo consistent oxidative stress events and did not exhibit morphological modifications or adverse reactions in mitochondrial membrane potential. Our findings highlight the possibility to use Neural Stem Cells plated on 3C-SiC substrate as clinical tool for lesioned neural areas, paving the way for future perspectives in novel cell therapies for neuro-degenerated patients.

## Introduction

Stem cells are increasingly attracting researchers’ attention thanks to their potential to differentiate into several lineages suitable for cell replacement therapies (e.g. cardiomyocytes, chondrocytes, endothelial cells, etc)^1–3^, representing a promising tool as neuronal cell sources for repairing or regeneration of the damaged nervous system^3,4^. Indeed, the adult neural tissue is characterized by an extremely limited self-repairing capacity. This issue justifies the search for new sources of cells in the treatment of post-traumatic and hereditary disorders as well as novel strategies or interventions applicable to neurodegenerative diseases. Despite the great potentiality, neural stem cell-based therapy has encountered technical and ethical restrictions for use in clinical practice, due to their limited expansion ability in culture and the difficulty in tracking their fate after *in vivo* implantation^5^.

Among the different types of currently available stem cells, neural stem cells (NSCs) have been widely applied as suitable cells for neuro-regeneration. NSCs can be differentiated from different sources, including dental pulp stem cells (DPSCs)^6^. Previous research showed the innate neurogenic potential of DPSCs, which are derived from the neural crest^7^.

Another type of interesting cells for their ability to promote axonal regeneration and functional restoration in the lesioned neural areas are the Olfactory Ensheathing Cells (OECs). OECs are a type of glial cells showing phenotypic properties with both Schwann Cells (SCs) and astrocytes, and own peculiar characteristics: plasticity and ability to secrete several Growth Factors (GFs), such as neurotrophins and neuregulins^8^.

Recently, OECs and DP-NSCs have been recognized as an interesting alternative source of stem cells for cellular transplantations strategies^8,9^ and the management of neurodegenerative diseases and Spinal Cord Injury, one of the most devastating forms of injury leading to disability and death^9^. Moreover, the combination of stem cells and nanotechnologies seem to be a promising approach for the development of clinical translatable cell-based therapies enhancing neural repair.

Following this standpoint, nanotechnology and regenerative medicine strategies represent a future perspective for the development of novel therapies that would reach from bench to bedside to serve the neuro-degenerated patients.

In this context, many candidate semiconducting materials for biotechnological applications have been investigated for biocompatibility and sensing potentiality. The main concern is to find a suitable material that produces low or no adverse effect when grafted in the body that can be implanted for long term and is capable of interfacing with electronic devices. In the field of semiconductors, Silicon (Si) has been the preferred substrate material for micro-devices due to its low cost and ready availability. However, it presents several drawbacks that limit its use in biomedical applications, specifically a low interaction rate with the body and a short period of stability when used *in vivo*. Especially for permanent implanted devices (such as glucose sensors, brain-machine-interface devices, smart bone and organ implants), a more performing material is required that is not rejected by the body and not recognized as a foreign material^10^. Silicon Carbide (3C-SiC) has been proven to be a good substrate for this purpose, being bio- and hemo-compatible, and usable for the manufacture of implantable devices^10,11^.

3C-SiC is a wide bandgap semiconductor (Eg = 3.2 eV) with extraordinary chemo-physical properties. It is both extremely stable and inertness, and it is able to work at high frequency, high-temperature, and in harsh environments, with better performances than other silicon-based devices. It is a polymorphic material with more than 200 different known polytypes, among which the most used are the cubic 3C-SiC and the hexagonal 4H-SiC^12,13^. 4H-SiC is being used for high power electronic devices, while 3C-SiC has a surface with chemical-physical properties that makes it highly suitable for biotechnological applications. Previously, the main drawbacks of 3C-SiC was the high cost and the low quality of the substrate (compared with Si). Nevertheless, the cost has considerably decreased in recent years, and material quality has achieved very high standards, therefore implantable radiofrequency (RF) devices for real time and *in vivo* measurements of the glucose and neuronal interface devices have already been manufactured^14,15^. 3C-SiC is thus considered a suitable bridge between internal neural networks and external electronic devices^16^.

The aim of the present work was to test the biocompatibility of Si and 3C-SiC in an *in vitro* model of human neurons derived from dental pulp mesenchymal stem cells, and in OECs derived from mouse olfactory bulbs.

## Results

### Material characterization

**Figure 1:**
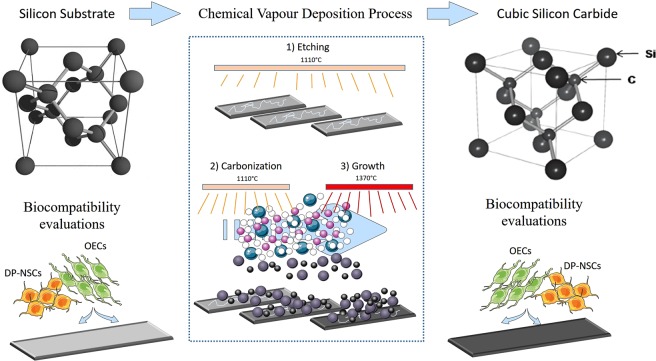
Synthesis of 3C-SiC wafers. Silicon Carbide wafers were artificially synthetized through epitaxial chemical vapor deposition process in a horizontal hot-wall reactor using silicon wafers as substrate. The process in composed of three main steps: “Etching” and “carbonization” steps were performed at 1100 °C, while “growth” step was performed at 1370 °C. Trichlorosilane (green dots) and ethylene (pink dots) were used as silicon (gray dots) and carbon (black dots) precursors respectively carried by hydrogen (with dots). After that, DP-NSCs and OECs were seeded on Si and 3C-SiC wafers to evaluate the biocompatibility. This figure was realized with elements from Servier Medical Art (www.servier.fr/servier-medical-art).

**Figure 2:**
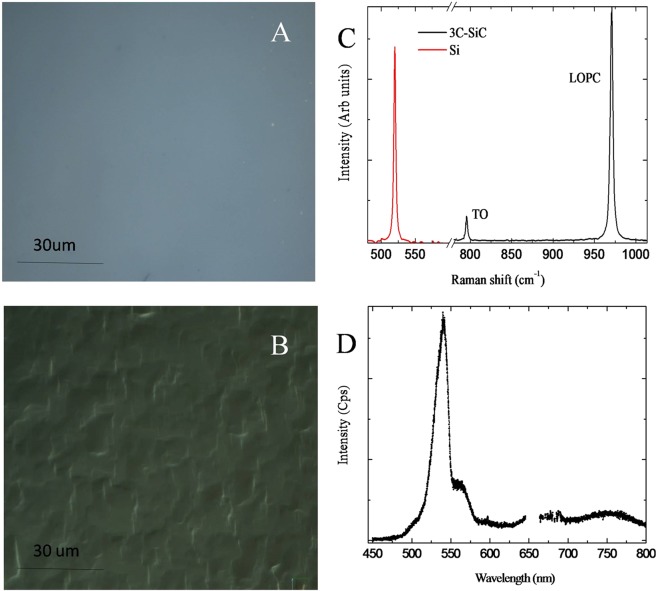
Characterization of Si and 3C-SiC wafers. Optical images of Silicon (**A**) and 3C-Silicon Carbide (**B**) surfaces. Si (red) and 3C-SiC (black) Raman spectra (**C**). In the same figure TO and LOPC peaks are labeled. Photoluminescence spectra of 3C-SiC (**D**).

Silicon Carbide wafers were artificially synthetized through epitaxial chemical vapor deposition process from silicon as substrates (Fig. 1). Optical microscopy of 3C-SiC and Si surface is shown in Fig. 2A,B, respectively. Perfectly flat surface is observed for Si substrate, while undulation is apparent in 3C-SiC. The Raman spectra of the 3C-SiC (black) and the Si (red) samples is shown in Fig. 2C. A sharp peak at 520 cm^−1^ is apparent in the Raman spectra, the position and the Full Width at Half Maximum (FWHM) indicate the high crystalline quality of the Si used. 3C-SiC Raman spectra is composed by TO and LOPC vibration at 769 and 972 cm^−1^, respectively. The information on the crystal quality, orientation, stress and donor density of the synthesized material can be deduced by the shape and position of the Raman peaks of 3C-SiC. In our experimental configuration (in back scattering configuration) TO vibration is not allowed (for 001 crystal direction) because of the selection rules. Nevertheless, the presence of small amount defects gives the peak observed in Fig. 2C. Unfortunately, it is not possible to have a quantitative measure of the amount of defect that presumably are stacking faults. The TO spectral position gives information on the wafer residual stress induced by the Si substrate and by defects. Spectral shift of the TO is linearly related to the lattice compression. By using the following formula: W_TO_ = 796.5–3734 (Da)/a^17^, a negligible tensile stress was observed due to the high quality of the sample. Donor density was measured by accurately fitting the LOPC peak with a suitable model as reported elsewhere^18^. Fitting procedure allows us to estimate a donor concentration of less than 1 × 10^17^cm^−3^.

In Fig. 2D, the 3C-SiC photoluminescence band at 540 nm (2.3 eV) is shown. The presence of this band is related to the high quality of the crystal and is associated to both the band-to-band transition and the nitrogen doping emission. Small band related to the presence of defects is apparent at the 750 nm. Si substrate has a negligible luminescence intensity and it is not shown in Fig. 2D.

### Neuronal differentiation from DPSC

**Figure 3:**
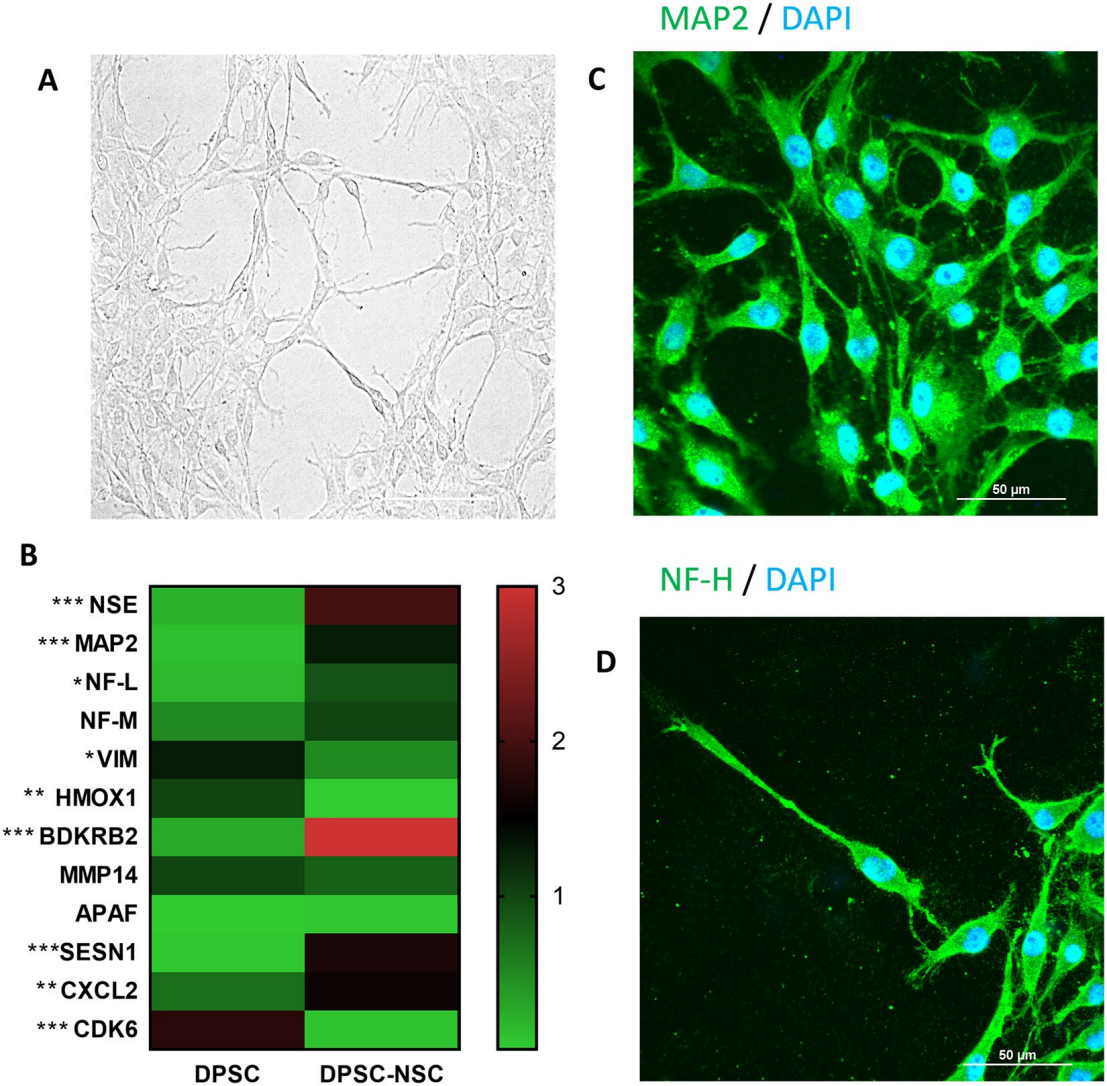
Differentiation of Dental Pulp Stem Cells into Neural Stem Cells. (**A**) Representative photomicrograph of neuronal-like stem cells derived from DPSC in bright field taken from randomly selected slides scanned by a Nikon Ti Eclipse inverted microscope (scale bar 50 μm). (**B**) Gene expression changes following differentiation. Quantitative real-time PCR assay was performed to assess gene expression changes after 16 days of RA-treatment. mRNA levels were normalized to the amount of β-actin mRNA and represented in a heatmap (*P < 0.05 and ***P < 0.001 as determined by two-way ANOVA followed by Sidack post hoc test). (**C**,**D**): representative photomicrographs of DP-NSCs show the fluorescent distribution of neuronal marker NF and MAP2. Nuclei were counterstained with DAPI (scale bar 50 μm).

In order to differentiate DPSCs in neurons-like cells, a treatment with retinoic acid for 15 days was performed. Cells at endpoint of differentiation show a classical neuronal morphology with very thin and long cytoplasmic processes, axon and dendrite presence, and perikaryon (Fig. 3A). To assess neuronal differentiation, we measured the expression of neuronal markers *NEUROFILAMENTS LIGHT CHAIN* (*NF-L*), *NEUROFILAMENT MEDIUM CHAIN* (*NF-M*), *MICROTUBULE-ASSOCIATED PROTEIN 2* (*MAP2*) and *NEURONAL SPECIFIC ENOLASE* (*NSE*) by qRT-PCR assays. In addition to these, we extended the analysis to eight additional genes whose differential expression has been found in neurons from differentiated DPSCs^19^: *VIMENTIN* (*VIM*), *HEME OXYGENASE 1* (*HMOX1*), *BRADYKININ B2 RECEPTOR* (*BDKRB2*), *MATRIX METALLOPEPTIDASE 14* (*MMP14*), *APOPTOTIC PROTEASE ACTIVATING FACTOR-1* (*APAF-1*), *SESTRIN 1* (*SESN1*), *C-X-C MOTIF CHEMOKINE LIGAND 2* (*CXCL2*) and *CYCLIN DEPENDENT KINASE 6* (*CDK6*). These genes are known to be involved in different biological processes, such as cell cycle progression and regulation (decreased during neural differentiation) or nervous system development and regulation of neurotransmitters (increased during neural differentiation). Our results confirm a significant up-regulation of *NSE*, *MAP2*, *NF-L*, *BDRKB2*, *SESN1* and *CXCL2*, and a down-regulation of *VIM*, *HMOX1*, and *CDK6* in neuronal cells differentiated from DPSC (Fig. 3B).

Moreover, the expression of neuronal markers in DP-NSCs was confirmed by immunostaining of *NF-H* and *MAP2* (Fig. 3C,D).

### OEC characterization

**Figure 4:**
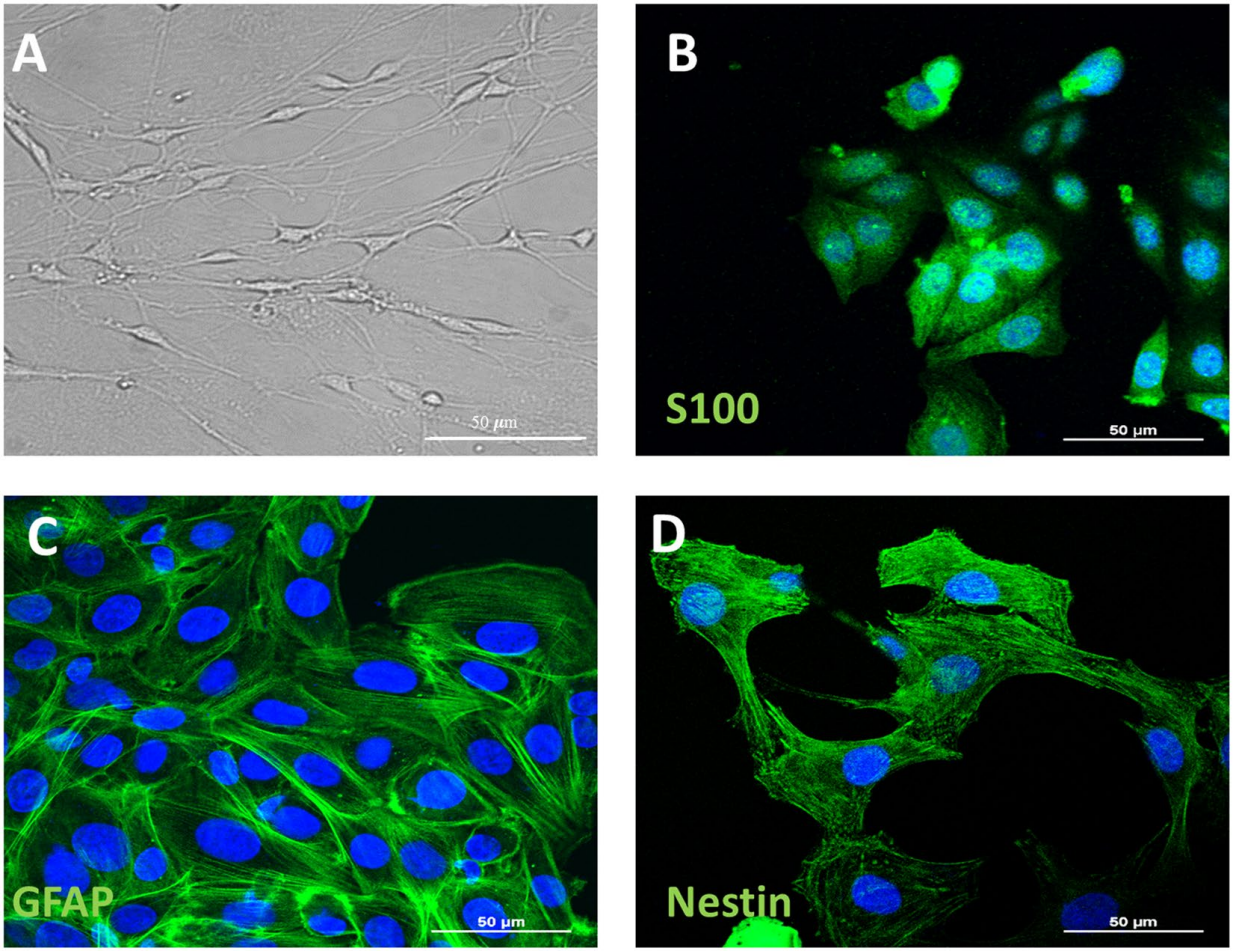
Characterization of Olfactory Ensheathing Cells. (**A**) Representative photomicrograph of OECs in bright field taken from randomly selected slides scanned by a Nikon Ti Eclipse inverted microscope (scale bar 50 μm). (**B**–**D**) Expression and distribution of specific neural markers, (S100, GFAP and Nestin) in OECs. Nuclei were counterstained with DAPI (scale bar 50 μm).

In Fig. 4A, OECs show an elongated shape with a dense network in bright-field. Immunohistochemical analysis of OECs was performed for the following markers: S-100, GFAP, and nestin. S-100 is a specific marker for OECs (Fig. 4B), GFAP highlights the differentiation of OECs towards astroglial phenotype (Fig. 4C), while nestin is a stem cell marker whose expression shows that OECs acquired stem cell features (Fig. 4D).

### Cell morphology by scanning electron microscopy

**Figure 5:**
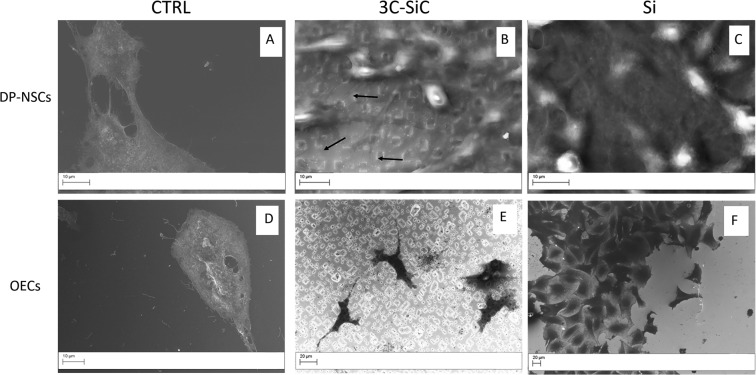
Investigation of DP-NSCs and OECs morphology by scanning electron microscopy. SEM micrograph shows the round morphology of DP-NSCs grown on 3C-SiC (**B**) and Si (**C**) compared with flat morphology of those growing on glass (control **A**). On 3C-SiC, DP-NSCs exhibit a high number of thin filopodia (arrows in **B**) compared to Si. Compared with flat control cells (**C**), on 3C-SiC (**D**) and on Si (**E**) OECs acquire a rod-shaped morphology with long cellular extensions visible on 3C-SiC compared with Si. Scale bar 10 μm (**A**–**D**) and 20 μm (**E**,**F**).

Using Scanning Electron Microscopy (SEM), we analyzed morphology and adhesive structures of DP-NSCs and OECs growing on Si and 3C-SiC (Fig. 5). After 48 hours of incubation on glass, both control DP-NSCs and OECs exhibited flatter and well spread morphology with, in the case of DP-NSCs, numerous adhesive structures (filopodia and lamellipodia). On Si and 3C-SiC, DP-NSCs acquired a rounded form, which is typical of the first phases of substrate adhesion^20^, and large adhesion structures. However, DP-NSCs grown on 3C-SiC exhibited a high number of thin filopodia (arrows in Fig. 5B) and large lamellipodia whose ability to form intercellular connections did not seem to be influenced by the presence of cubic crystals. On Si and 3C-SiC, OECs acquired a rod-shaped morphology with long cellular extensions when seeded on 3C-SiC compared with those growing on Si (Fig. 5E,F).

### Si/3C-SiC substrate influence on mitochondrial activity

**Figure 6:**
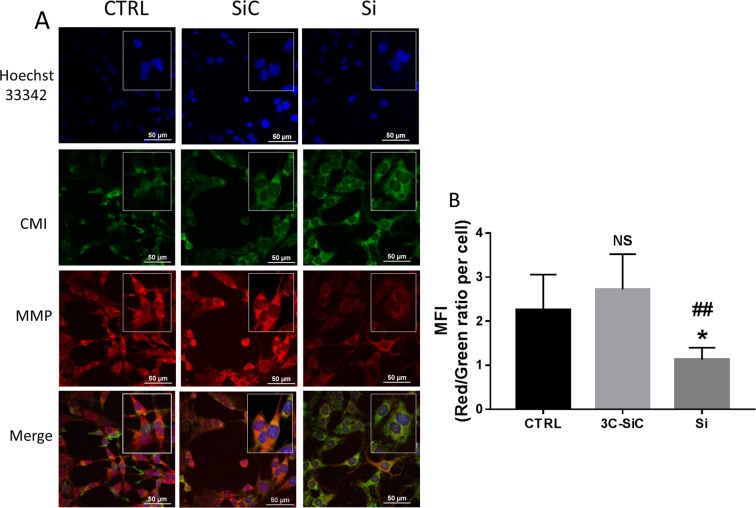
Effects of Si and 3C-SiC on mitochondrial membrane potential and cell membrane integrity of DP-NSCs. (**A**) Representative results were taken from randomly selected slides scanned by a Nikon Ti Eclipse inverted microscope. MitoHealth (red) stains stable mitochondrial membrane potential, DEAD Green (green) stains deadly cells, Hoechst 33342 (blue) stains nuclei. A merged image for all experimental condition was created with NIS Elements software. (scale bar 50 μm). (**B**) Mean Fluorescence Intensities (MFI) ratio of MitoHealth (red) and DEAD Green (green) per cell. Differences in MFIs were not statistically significant between glass-plated control cells and those plated on 3C-SiC for 48 hours (3C-SiC *vs* Si, P < 0.005; control group *vs* Si, P < 0.05; differences in fluorescence intensity were not significant statistically between 3C-SiC and control group).

**Figure 7:**
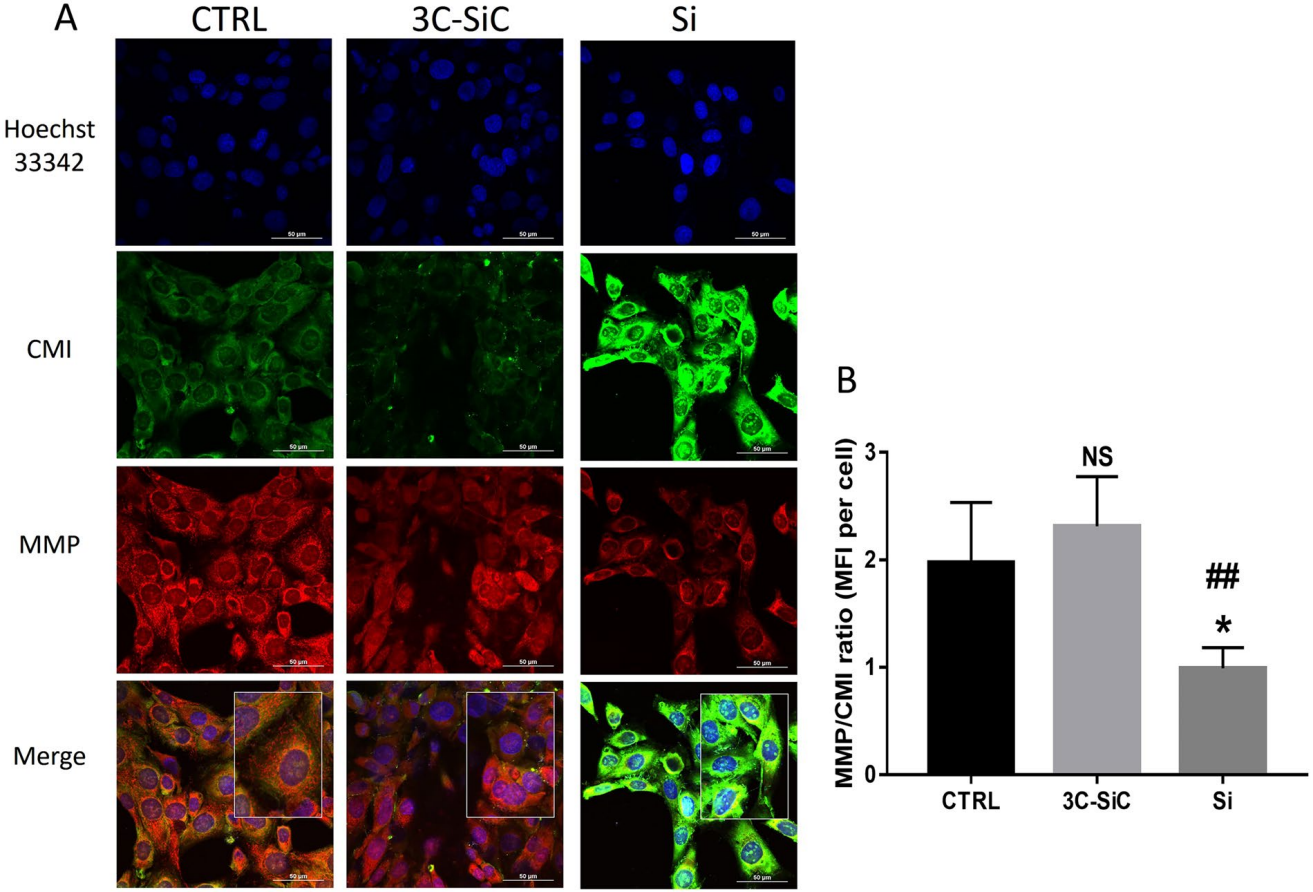
Effects of Si and 3C-SiC on mitochondrial membrane potential and cell membrane integrity of OECs. (**A**) Representative results were taken from randomly selected slides scanned by a Nikon Ti Eclipse inverted microscope. MitoHealth (red) stains stable mitochondrial membrane potential, DEAD Green (green) stains deadly cells, Hoechst 33342 (blue) stains nuclei. A merged image for all experimental conditions was created with NIS Elements software (scale bar 50 μm). (**B**) Mean Fluorescence Intensities (MFI) ratio of MitoHealth (red) and DEAD Green (green) per cell. Differences in MFIs were not statistically significant between glass-plated control cells and those plated on 3C-SiC for 48 hours (3C-SiC *vs* Si, P < 0.005; control group *vs* Si, P < 0.05; differences in fluorescence intensity were not significant statistically between 3C-SiC and control group).

**Figure 8:**
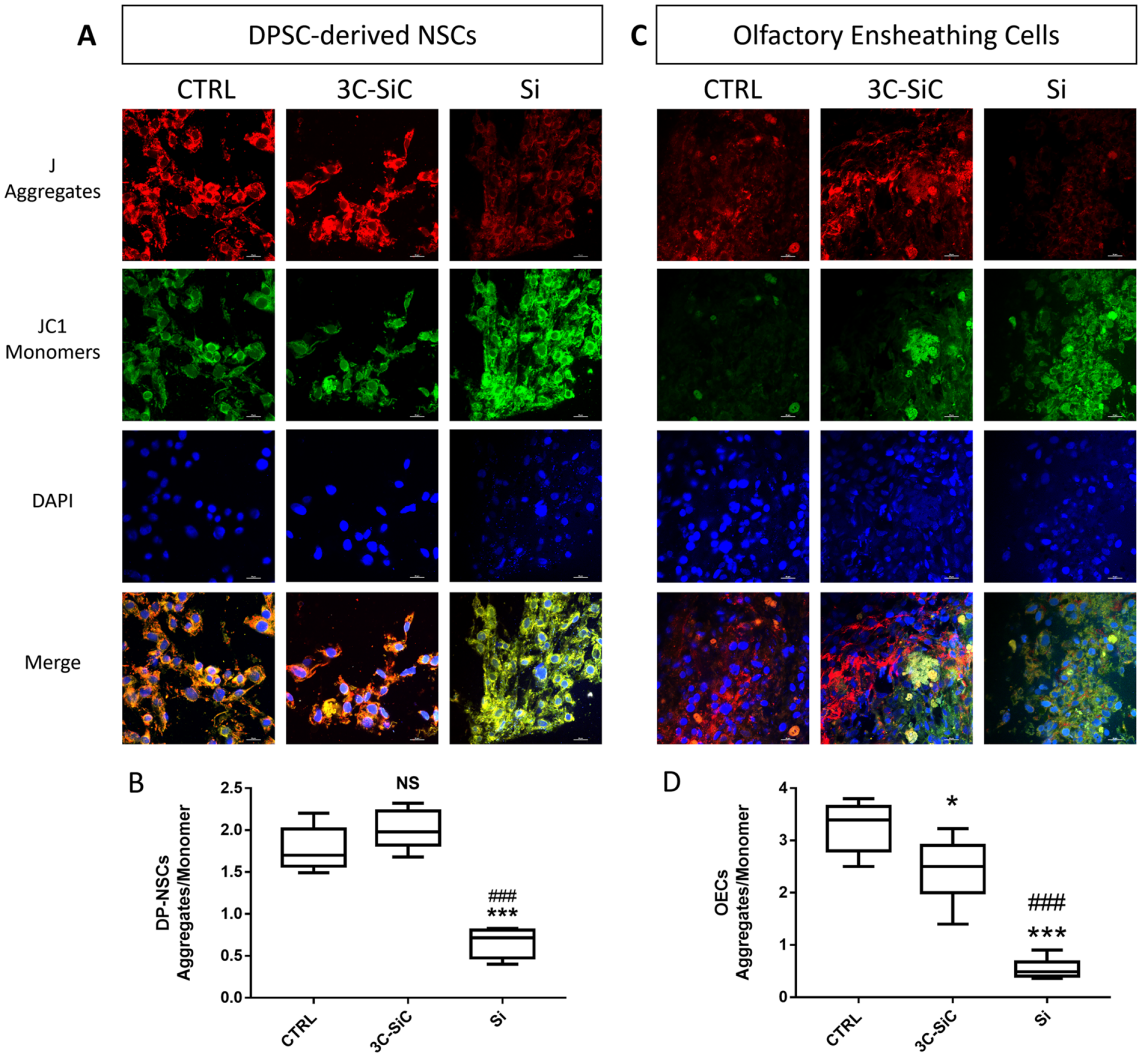
Effects of Si and 3C-SiC on mitochondrial membrane potential of DP-NSCs and OECs. Representative microphotograph of DP-NSCs (Panel A) and OECs (Panel C) stained with JC-1 after 48 hours of exposure to 3C-SiC and Si. Mitochondrial J-aggregates in red; cytosolic JC-1 monomers in green; DAPI (blue) stain nuclei. A merged image for all experimental condition was created with NIS Elements software (scale bar 20 μm). Mean Fluorescence Intensities (MFI) ratio of J-aggregates (red) and JC-1 monomers (green) per cell were calculated for DP-NSCs (Panel B; ***P < 0.001 Si *Vs* Control and ^###^P < 0.001 Si *Vs* 3C-SiC as determined by one-way ANOVA followed by Tukey post hoc test) and OECs (Panel D; *P < 0.05 3C-SiC *Vs* Control, ***P < 0.001 Si *Vs* Control and ^###^P < 0.001 Si *Vs* 3C-SiC as determined by one-way ANOVA followed by Tukey post hoc test).

To investigate the different biocompatibility of Si and 3C-SiC on DP-NSCs and OECs, we used a double stains-based commercial kit to simultaneously measure two important cell-health parameters: mito-toxicity and cytotoxicity. In particular, the MitoHealth red stain accumulates in live cells mitochondria proportionally to the mitochondrial membrane potential, while the Image-iT®DEAD Green™ viability stain measures cytotoxicity. Representative images show how the exposure to Si decreased the mitochondria membrane potential and produced cytotoxicity after 48 hours of incubation, while mito-toxicity and cytotoxicity of 3C-SiC-exposed cells were similar to controls in both DP-NSCs (Fig. 6A) and OECs (Fig. 7A). Fluorescence intensity ratios of red/green channels, normalized by blue channel (DAPI staining), show a reduced biocompatibility of Si when compared to 3C-SiC (3C-SiC *vs* Si, P < 0.005; control group *vs* Si, P < 0.05; differences in fluorescence intensity were not significant statistically between 3C-SiC and control group) in both DP-NSCs (Fig. 6B) and OECs (Fig. 7B). To further confirm the mitotoxicity induced by Si wafers in DP-NSCs and OECs, the mitochondrial membrane potential (MMP) reduction was analyzed through the use of JC-1. As shown in Fig. 8, 3C-SiC did not impair MMP in DP-NSCs and slightly depolarized mitochondria in OECs (*P < 0.05). Conversely, Si wafers altered mitochondrial potential both in DP-NSCs and OECs (***P < 0.001), given the green fluorescence emitted by JC-1 monomers accumulated in the cells cytoplasm as reported in Fig. 8A,B for DP-NSCs and 8C-D for OECs.

### Si/3C-SiC substrate influence on cell death

During apoptosis cells exhibit phosphatidylserine (PS), by an ATP-dependent flippase mechanism, even before the loss of permeability. In order to evaluate the apoptosis induction rate of Si/3C-SiC surfaces, we marked simultaneously the cells with FITC-conjugated Annexin V and Propidium Iodide (PI). Annexin V is a protein able to bind selectively PS in presence of calcium ion, while IP is a fluorescent intercalating agent not permeant to live cells. In this way, we distinguish alive (with intact cytoplasmic membrane), apoptotic or necrotic cells.

**Figure 9:**
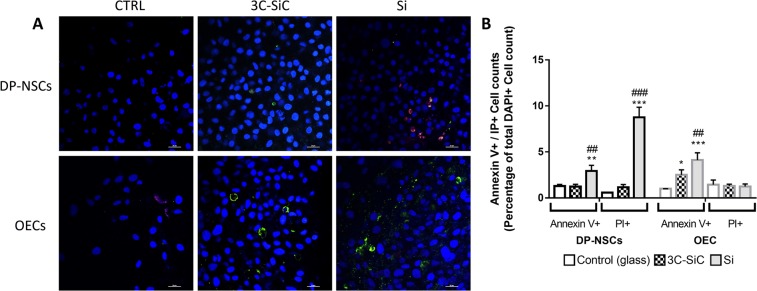
Effects of Si and 3C-SiC on apoptotic cell death of DP-NSCs and OECs. (**A**) Representative merged microphotograph of DP-NSCs and OECs marked with Annexin V and PI after 48 hours of exposure to 3C-SiC and Si. Cells can be divided into alive (with intact cytoplasmic membrane), apoptotic (membrane green fluorescence for Annexin V) or necrotic (nuclear red fluorescence for PI). DAPI (blue) stains nuclei (scale bar 20 μm). (**B**) Annexin V+ and PI+ cell count is reported as percentage of total DAPI + cells per field. All cell counts for all experimental condition were obtained with NIS Elements software (*P < 0.05 3C-SiC *Vs* Control, ***P < 0.001 Si *Vs* Control and ^###^P < 0.001 Si *Vs* 3C-SiC as determined by one-way ANOVA followed by Tukey post hoc test).

Representative images (Fig. 9) show that the exposure to Si triggers significantly apoptosis and necrosis in DP-NSCs model, increasing both the number of Annexin V+ (+2.9% ± 0.6 relative to total DAPI+ cells per field; **P < 0.005) and PI+ cells (8.7% ± 1.1 relative to total DAPI+ cells; ***P < 0.0001).

Instead, apoptosis was revealed in OECs seeded on both 3C-SiC wafer (+2.5% ± 0.5 relative to total DAPI+ cells; *P < 0.05) and Si wafer (+4.1% ± 0.8 relative to total DAPI+ cells; ***P < 0.0001). No necrotic (PI+) OECs were observed (Fig. 8A,B).

## Discussion

Semiconductor-based biomaterials represent a domain for important biomedical applications, from heart stent coatings^21^ to brain implants^22,23^, tissue implant scaffolds^10^ and *in vivo* biosensors^24^. The main concern is to find a suitable material that produces low or no adverse effect when grafted in the body, and it can be implanted for long term and is capable of interfacing with electronic devices.

In brain, semiconductor-based biomaterials are used for neural prosthetics, with stimulating and recording electrodes, or as scaffold to enhance neuronal repair^25^. Stimulation-based electrodes are used to restore vision^26^, hearing^27^ or alleviate the symptoms of Parkinson’s disease^28^. Recording based prosthetics hold great promise for movement restoration^29^, such as in patients affected by traumatic brain injury, stroke or amyotrophic lateral sclerosis. Semiconductor-based biomaterials are also used in neural tissue engineering as scaffold^30^ to control axonal growth through the lesion site^31,32^, improve cell’s survival rate and maintenance of grafted cells^33–36^ or drive the host cell migration (e.g., Schwann cells and astrocytes)^37^. Recent investigations reported that transplantation of NSCs, SCs and OECs showed encouraging effects on neural regeneration and functional recovery in some neurodegenerative diseases^38^. Thus, the study of neural cell behavior, such as adhesion, viability and biocompatibility, to different semiconductor-based surfaces is fundamental to develop new novel biomedical devices, since different studies reported that biomaterial supports can serve multiple functions in transplantation approaches^39^.

Recently, silicon carbide has attracted the attention of researchers due to its higher biocompatibility compared to Si, as assessed through preliminary *in vitro*^40^ and *in vivo*^41^ studies. The present work explores the biocompatibility of Si and 3C-SiC with neural stem cells (DP-NSCs and OECs). Our results demonstrate the higher biocompatibility of 3C-SiC compared to Si, based on morphological, mitochondrial and viability parameters. Morphologically, adhesion to 3C-SiC altered the number and the thickness of DP-NSCs filopodia and lamellipodia. Differentially, OECs acquired a rod-shaped morphology with long cellular extensions. Adhesion to silicon-based substrates influenced also the mitochondrial membrane potential as assessed by MitoHealth and JC-1 stains, showing that Si is extremely chemically reactive leading to bioenergetics dysfunction of mitochondria. Finally, neural cells grown on 3C-SiC were more viable respect to those seeded on Si wafers, since the latter triggered apoptotic and necrotic phenomena in DP-NSCs and increase OECs apoptosis.

To date, this is the first report assessing the higher biocompatibility of 3C-SiC compared to Si with neural stem cells. Further studies are necessary to investigate the long-term compatibility between 3C-SiC substrate and neural stem cells, and the potential of this substrate as clinical tool for monitoring, recording and stimulate neuronal activity in lesioned brain areas.

## Materials and Methods

### 3C-SiC Growth

The 3C-SiC films were realized with an epitaxial chemical vapour deposition process, in a horizontal hot-wall reactor in LPE factory on silicon substrate. The hetero-epitaxy is realized on 6 inches on-axis (001) Si substrate. Trichlorosilane, ethylene and hydrogen were used as silicon and carbon precursors and gas carrier, respectively. The processes were realized in a low-pressure regime (10^4^ Pa) at a temperature of 1370 °C. Several different steps constituted the entire deposition process. Three main steps can be mentioned: “etching”, “carbonization” and “growth”. “Etching” and “carbonization” steps were performed at lower temperature 1100 °C, while the growth temperature was set at 1370 °C. Growth rate was changed in the ranges 3 to 30 μm/h. After the growth, the precursor flows were stopped, and the temperature was decreased to 200 °C in an Argon environment^42–44^.

Si wafer used as substrate for the 3C-SiC growth was utilized in stem cell experiments. It is a standard 300 μm thick and 10 ohm/cm resistivity N-doped wafer commonly used in microelectronic field.

### 3C-SiC and Si optical characterization: Optical microscopy, Raman and Photoluminescence

In order to characterize the 3C-SiC layer and Si substrate we performed Optical microscopy, UV and micro Raman analysis. UV Raman spectra were collected using an HR800 integrated system by Horiba Jobin Yvon working in back-scattering configuration. The excitation source was supplied by a He-Cd laser with a wavelength of 325 nm. Power ranges from 1 to 10 mW (with a power density of about 0.5 to 5 kW/cm2, respectively). Confocal optics provided with a dichroic mirror for 325 nm light is used and laser light was focused via 40X objective onto the sample. The emitted light was dispersed by a 1800 or 300 grooves/mm-kinematic grating for Raman or photoluminescence measurements.

### Dental pulp stem cell isolation

DPSCs were obtained from five extracted teeth of five patients^45^. This study has been reviewed and approved by an Institutional Review Board (IRB) of Azienda Ospedaliero-Universitaria “Policlinico-Vittorio Emanuele”, Catania (n.92/2015/CA). Experiments were approved by an ethical committee for medical research and performed in accordance with ethical standards. Informed consents were obtained from patients for the use of their samples and for the access to medical records for research purposes. Briefly, to isolate the pulp tissue freshly extracted teeth were splayed to remove the pulp tissue, then chopped into small fragments of 1 mm, and treated for the enzymatic digestion with 3 mg/ml collagenase type I (Gibco Invitrogen, Carlsbad, CA) for 1 h at 37 °C. The tissue pellet was suspended in Dulbecco’s Modified Eagle’s Medium (DMEM) added with penicillin (100 U/ml), streptomycin (100 mg/ml) and 15% Fetal Bovine Serum (FBS). DPSC were cultured at 37 °C in a humidified atmosphere of 5% CO_2_ and the medium was replaced every 3 days.

### Cell culture and neuronal differentiation

The neural differentiation from dental pulp stem cells was obtained as reported in our previous study^5^. Briefly, cells were cultured for 6 days *in vitro* (DIV) in the basic medium (DMEM), subsequently the medium was added with the following neural induction cocktail: 0.5 mM Isobutyl Methyl Xanthine (IBMX), 20 ng/ml human Epidermal Growth Factor (hEGF) 1 mM dibutyrylcAMP (dbcAMP), 10 ng/ml Nerve Growth Factor (NGF) and 10 ng/ml Brain-Derived Neurotrophic Factor (BDNF), 40 ng/ml basic Fibroblastic Growth Factor (bFGF), (all reagents were purchased from Invitrogen, Milan, Italy). Obtained neuronal differentiation, cells were grown in culture for 15 days in DMEM/FBS added with 10 µM retinoic acid^21^.

### OECs cultures

OECs were isolated from 2-day old mouse pups (P2) olfactory bulbs (provided by Envigo RMS s.r.l. Italy). All the experimental procedures were carried out according to the Italian Guidelines for Animal Care (D.L. 116/92 and 26/2014), which are in compliance with the European Communities Council Directives (2010/63/EU) and were approved by the Ethical Committee at the University of Catania (Italy).

Briefly, pups were decapitated and the bulbs were removed and placed in cold (+4 °C) Leibowitz L-15 medium (Sigma-Aldrich, Milan, Italy). Then, they were digested in collagenase and trypsin mixture. Trypsinization was stopped by adding DMEM supplemented with 10% FBS. Cells were re-suspended and plated in flasks fed with DMEM/FBS. To reduce the number of dividing fibroblasts the antimitotic agent, cytosine arabinoside (10^−5^M), was added 24 h after initial plating. In the last passage, OECs were plated on 25 cm^2^ flasks and cultured in DMEM/FBS supplemented with bovine pituitary extract^46^. Cells were incubated at 37 °C in complete medium and fed twice a week.

### Cell growth on Si and 3C-SiC substrates

Both DP-NSCs and OECs were grown on 3C-SiC and Si rectangular surfaces at a final density of 0.3 × 10^3^ cells/cm^2^ for 48 h before the immuno-cytochemical procedures and mitochondrial Health assay. Control cells were plated on glass coverslip, without Si and 3C-SiC substrates.

### Scanning electron microscopy

Both DP-NSCs and OECs were cultured on 3C-SiC and Si for 48 h. After incubation, cells were washed with PBS and fixed with 2% glutaraldehyde for 1 h at room temperature. Cells were dehydrated in graded ethanol solutions from 50 to 100%. SEM images were obtained by using a Zeiss Supra-55 VP field emission scanning electron microscope (FEG-SEM). Control samples on glass were sputtered with gold.

### Immunofluorescent assay

Immunofluorescent assays were performed in differentiated DP-NSCs and in OECs to confirm the expression of different markers. For DP-NSCs we investigated the neuronal markers heavy chain neurofilament (NF-H) and microtubule associated protein – type 2 (MAP2). For OECs we analyzed the following markers: Nestin, Glial Fibrillaric Acid Protein (GFAP), and S100. All cells, seeded on coverslips, were fixed with 4% paraformaldehyde for 15 min and then permeabilized with 0.1% Triton-X in PBS for 10 min. The following primary antibodies were incubated overnight at 4 °C: mouse monoclonal anti-NF-H antibody (1:200; Abcam, Cambridge, UK), mouse monoclonal anti-MAP2 antibody (1:1000; Merck Millipore, Billerica, Massachusetts, USA), mouse monoclonal anti-S-100β antibody (1:100; Sigma), rabbit polyclonal anti-nestin antibody (1:200; AbCam), rabbit polyclonal anti-GFAP (1:1000; Dako). A goat anti-mouse or a goat anti-rabbit FITC-conjugated secondary antibodies (1:100; Jackson ImmunoResearch, West Baltimore Pike, Pennsylvania, USA) were used for 1 h at room temperature in dark. Coverslips were then washed three times in PBS, mounted with glycerol mounting medium with DAPI (Abcam, Cambridge, UK) on glass microscope slides, and analyzed using confocal laser microscopy (A1; Nikon, Tokyo, Japan) through a Plan Apochromat lambda 60X/NA1.4 oil immersion lens (Nikon, Tokyo, Japan).

### qRT-PCR assay

Total mRNA was isolated with TRIzol reagent (Thermo Fisher Scientific, Waltham, Massachusetts, USA) allowing manufacturer’s guidelines^22^. Qualitative and quantitative analysis of extracted RNA were performed with NanoDrop1000 spectrophotometer (Thermo Fisher Scientific, Waltham, Massachusetts, USA). A part of total RNA (4μg) were reverse transcribed with SuperScript™ III Reverse Transcriptase Kit (Thermo Fisher Scientific, Waltham, Massachusetts, USA). To perform quantitative RT-PCR assay, mRNA was converted into cDNA. To assess the neural differentiation achieved, quantitative RT-PCR was performed to investigate mRNA expression levels of different neuronal markers (*NF-L*, *NF-H*, *MAP2*, *NSE*, *VIM*, *HMOX1*, *BDKRB2*, *MMP14*, *APAF*, *SESN1*, *CXCL2* and *CDK6*). Primer pairs with relative amplification temperatures (AT) are reported in Table 1. PCR settings were established as follow: 1 cycle at 95°Cx2′; 50 cycles at 95°Cx5″, ATx10″, 72°Cx5″. The annealing temperature of actin depends on the target gene’s temperature. Each experimental condition was run in triplicate. Gene expression profiles were calculated with 2^ΔΔCt^ method for qRT-PCR and normalized to expression level of beta-actin^47^.

**Table 1:** List of used primers pairs.

Target	Primer F	Primer R	Expected bp (cDNA)	Annealing T (°C)
*ACT*	CTTCGCGGGCGACGAT	CACATAGGAATCCTTCTGACCC	103	*
*NEF-L*	TGTGCATGGACCACGCTTAT	TGCTAACCACCGAAGGTTCAA	152	60
*NEF-M*	CTTCCGCTCGCAGTCG	ATTCTGCTGCTCCAGGTAGT	277	55
*MAP2*	ATTGACAGCCAAAAGTTGAA	TCGAGCAGGTTGATGCTTCC	152	55
*NSE*	AGAAGCTGGACAACCTGATG	CTTCGCTGTTCTCCAGGATA	405	57
*VIM*	CTTCTCTGGCACGTCTTGAC	TCCTGGATCTCTTCATCGTG	94	55
*HMOX1*	AGTCTTCGCCCCTGTCTACT	CTTCACATAGCGCTGCATGG	113	60
*BDKRB2*	CCAGACGGAGAAGAAGGCCAC	AGCTGTTGCTATAGGCCACGTA	191	60
*MMP14*	GGTGGTCTCGGACCATGTCT	TGTGTGTGGGTACGTAGGTC	178	60
*APAF*	GAGGCTAAAGACCGTCTCCG	AGGAACTCTCCACAGGGACT	193	60
*SESN1*	CTGAAGAGCATCCAGGAAC	GCAGTAGATAGTGCTGAG	232	60
*CXCL2*	TGTCTCAACCCCGCATCG	TCTGGTCAGTTGGATTTGCCA	77	58
*CDK6*	GATGTGTGCACAGTGTCACGAAC	GTGGTTTTAGATCGCGATGCAC	205	58

*The annealing temperature of actin is the same of the target gene.

### Mitochondrial health assays

Mitotoxicity and cytotoxicity of both NSC-DPSC and OECs cultured on 3C-SiC and Si surface were analyzed by HCS Mitochondrial Health Kit (Thermo Fisher Scientific, Waltham, Massachusetts, USA) following manufacturer’s instruction. Briefly, after 48 hours of plating on Si and 3C-SiC, cells were stained with Mito-Health stain solution (in DMSO) and Image-iT® DEAD Green™ viability stain for 30 minutes under normal cell culture conditions. Then, cell medium was removed and 100 µl of counterstain/fixation solution (6 µl of Hoechst 33342 in 12 ml of 4% paraformaldehyde) was added to each well and incubated for 15 minutes at room temperature. After washing with PBS, 200 µl of PBS were added to each well, then 3C-SiC and Si wafers were analyzed using confocal laser microscopy (A1; Nikon, Tokyo, Japan) through a Plan Apochromat lambda 60X oil immersion lens (NA 1.4; Nikon, Tokyo, Japan).

Mitochondrial membrane potential (MMP) was investigated by the dual-emission potential-sensitive probe JC-1 (5,5′,6,6′ tetrachloro 1,1′3,3′ tetraethylbenzimidazolyl carbocyanine iodide - Sigma-Aldrich, Milan, Italy). JC-1, able to selectively penetrate in mitochondria, exists in monomeric form (emitting at 527 nm). In relation to the membrane potential, when excited at 490 nm JC-1 form aggregates (emitting at 590 nm). Therefore, the fluorescence changes reversibly from green to red-orange based on the mitochondrial membrane polarization. In viable cells with normal membrane potential, JC-1 accumulates in the mitochondrial membrane as aggregates emitting in an red-orange fluorescence, while in the cells with mitochondrial damaged it remains in the cytoplasm in a monomeric form, giving a green fluorescence^48^. Briefly, JC-1 probe was dissolved in cell medium at the final concentration of 10 μg/ml. Following 30 minutes’ incubation, cells were washed with PBS and mounted with glycerol mounting medium with DAPI (Abcam, Cambridge, UK) on glass microscope slides, and analyzed using confocal laser microscopy (A1; Nikon, Tokyo, Japan) through a Plan Apochromat lambda 60X/NA1.4 oil immersion lens (Nikon, Tokyo, Japan).

Mitochondrial membrane potentials of cells cultured on glass and Si and 3C-SiC substrates were extimated by the measure of red and green channel fluorescence intensities ratio per cell across 10 images for each experimental condition. The red and green fluorescence intensities per cell were normalized by the blue (DAPI) fluorescence intensity. Background subtraction was done for each image to normalize against background fluorescence. The data were reported as mean ratio fluorescence intensity (MFI) per cell and were obtained for each channel using NIS-Elements AR 4.60.000 software (Nikon, Tokyo, Japan).

### Live/Dead cell detection

In order to investigate *in situ* cell viability, morphological changes and the percentage of apoptotic cells were determined using FITC Annexin V/Dead Cell Apoptosis Kit (Thermo Fisher Scientific, Waltham, Massachusetts, USA) following manufacturer’s instruction slightly modified as follow: seeded cells were washed in PBS and directly incubated at room temperature for 15 minutes in dark with 5 μl of AnnexinV conjugate and 1 μl of 100 μg/ml of Propidium iodide (IP) dissolved in 100 μL of 1X Annexin Binding Buffer. after the incubation period, cells were washed with 1X Annexin Binding Buffer, fixed with paraformaldehyde, mounted with glycerol mounting *Medium with DAPI* (Abcam, Cambridge, UK) on glass microscope slides and analyzed using confocal laser microscopy (A1; Nikon, Tokyo, Japan) through a Plan Apochromat lambda 60X/NA1.4 oil immersion lens (Nikon, Tokyo, Japan).

The percentage of apoptotic cells were calculated counting Annexin V+, IP+ and DAPI+ cells thought the cell count function of NIS-Elements AR 4.60.000 software (Nikon, Tokyo, Japan).

### Statistical analysis

Data were reported as Mean ± S.E.M or Mean ± Standard deviation. To compare differences among groups one-way and two-way analysis of variance (ANOVA) was utilized. Statistical significance was assessed by the Tukey–Kramer post hoc test and the level of significance for all statistical tests was p ≤ 0.05.

## Data Availability

The datasets generated during and/or analysed during the current study are available from the corresponding author on reasonable request.
